# Genome characteristics reveal the impact of lichenization on lichen-forming fungus *Endocarpon pusillum* Hedwig (Verrucariales, Ascomycota)

**DOI:** 10.1186/1471-2164-15-34

**Published:** 2014-01-17

**Authors:** Yan-Yan Wang, Bin Liu, Xin-Yu Zhang, Qi-Ming Zhou, Tao Zhang, Hui Li, Yu-Fei Yu, Xiao-Ling Zhang, Xi-Yan Hao, Meng Wang, Lei Wang, Jiang-Chun Wei

**Affiliations:** 1 State Key Laboratory of Mycology, Institute of Microbiology, Chinese Academy of Sciences, Beijing 100101, China; 2 University of Chinese Academy of Sciences, Beijing 100049, China; 3 TEDA School of Biological Sciences and Biotechnology, Nankai University, Tianjin 300457, China

**Keywords:** Mycobiont, Phycobiont, Lichenization, Symbiosis, Symbiosis-related gene, Photosynthetic products

## Abstract

**Background:**

Lichen is a classic mutualistic organism and the lichenization is one of the fungal symbioses. The lichen-forming fungus *Endocarpon pusillum* is living in symbiosis with the green alga *Diplosphaera chodatii* Bialsuknia as a lichen in the arid regions.

**Results:**

454 and Illumina technologies were used to sequence the genome of *E. pusillum*. A total of 9,285 genes were annotated in the 37.5 Mb genome of *E. pusillum*. Analyses of the genes provided direct molecular evidence for certain natural characteristics, such as homothallic reproduction and drought-tolerance. Comparative genomics analysis indicated that the expansion and contraction of some protein families in the *E. pusillum* genome reflect the specific relationship with its photosynthetic partner (*D. chodatii*). Co-culture experiments using the lichen-forming fungus *E. pusillum* and its algal partner allowed the functional identification of genes involved in the nitrogen and carbon transfer between both symbionts, and three lectins without signal peptide domains were found to be essential for the symbiotic recognition in the lichen; interestingly, the ratio of the biomass of both lichen-forming fungus and its photosynthetic partner and their contact time were found to be important for the interaction between these two symbionts.

**Conclusions:**

The present study lays a genomic analysis of the lichen-forming fungus *E. pusillum* for demonstrating its general biological features and the traits of the interaction between this fungus and its photosynthetic partner *D. chodatii*, and will provide research basis for investigating the nature of its drought resistance and symbiosis.

## Background

A lichen is a symbiotic association of a fungus (*mycobiont*) and a photosynthetic partner (*photobiont*), which may be an alga (*phycobiont*) or a cyanobacterium (*cyanobiont*). In the association the fungus produces a *thallus*, or body, within which the photobionts are housed [1]. Around 20% of all Fungi and 40% of all Ascomycota are lichen-forming. Recent estimates of global diversity suggest that there are between 17,500 and 20,000 species [2].

Most lichens and isolated lichen-forming fungi grow extremely slow, but the lichen-symbiosis is a very successful association as lichens can survive in almost all adverse terrestrial conditions [3]. They are also famous for their particular secondary products, which are frequently used as antibacterial and antiviral compounds [4,5]. The lichen-forming fungi differ from non-lichenized fungi by their adaptations to symbiosis with photobiont [6]. This mutualistic association, as called lichenization, is one of the most important fungal lifestyles and the lichenization, considered by some researchers, has evolved many times in the phylogeny of fungi [7,8], and also some major fungal lineages may have derived from lichen symbiotic ancestors [9].

The principal problem about lichenization is the necessity of fungal propagules meeting a suitable photosynthetic partner for the resynthesis of the symbiosis [3,10]. The recognition step is complicated, involving many morphological and molecular changes. Scanning electron microscopy (SEM) has been used to investigate the changes in morphology during the early resynthesis of the lichen thallus [11-13]. However, few studies have explored the resynthesis events in lichen using molecular tools. Previous studies suggest that the mycobiont-derived lectins (sugar-specific, cell agglutinating proteins) may play a key role in recognition [14-17] between both symbionts.

The interdependent relationship between mycobiont and photobiont is the foundation of lichenization, which required for both symbionts to maintain each other. In the lichen thallus, the photobiont provides its mycobiont with photosynthetic products [18,19], previous reports showed that lichen-forming fungi absorb polyol (ribitol, sorbitol, and erythritol) or glucose from algae or cyanobacteria, respectively [20,21], and in most green algae lichens, the hyphae of the mycobionts wrap tightly around photobiont cells, thereby protecting the photobiont cells from a range of biotic and abiotic stress, including drought, high light and mechanical damage. The protection from light-injury is associated with secondary metabolic substances, such as melanins, produced by lichen-forming fungi [22]. However, little is known about the signals and mechanisms that lead to symbiotic recognition and maintenance in lichen, although it can be predicted that some metabolic products and macromolecules are essential.

The whole life cycle of lichen is rarely observed in the laboratory or in nature [11,13]. The detail of lichenization, whereby a lichen-forming fungus contacts with a compatible photosynthetic partner, recognizes it and captures it, has become a hot topic in recent studies [23,24]. *Endocarpon pusillum* is the most successful cases on artificial resynthesis of the fertile (perithecia-bearing) thalli from isolated mycobiont and phycobiont [12], and it will be very useful to reveal the origin of symbiosis between fungi and photosynthetic organisms.

**Figure 1:**
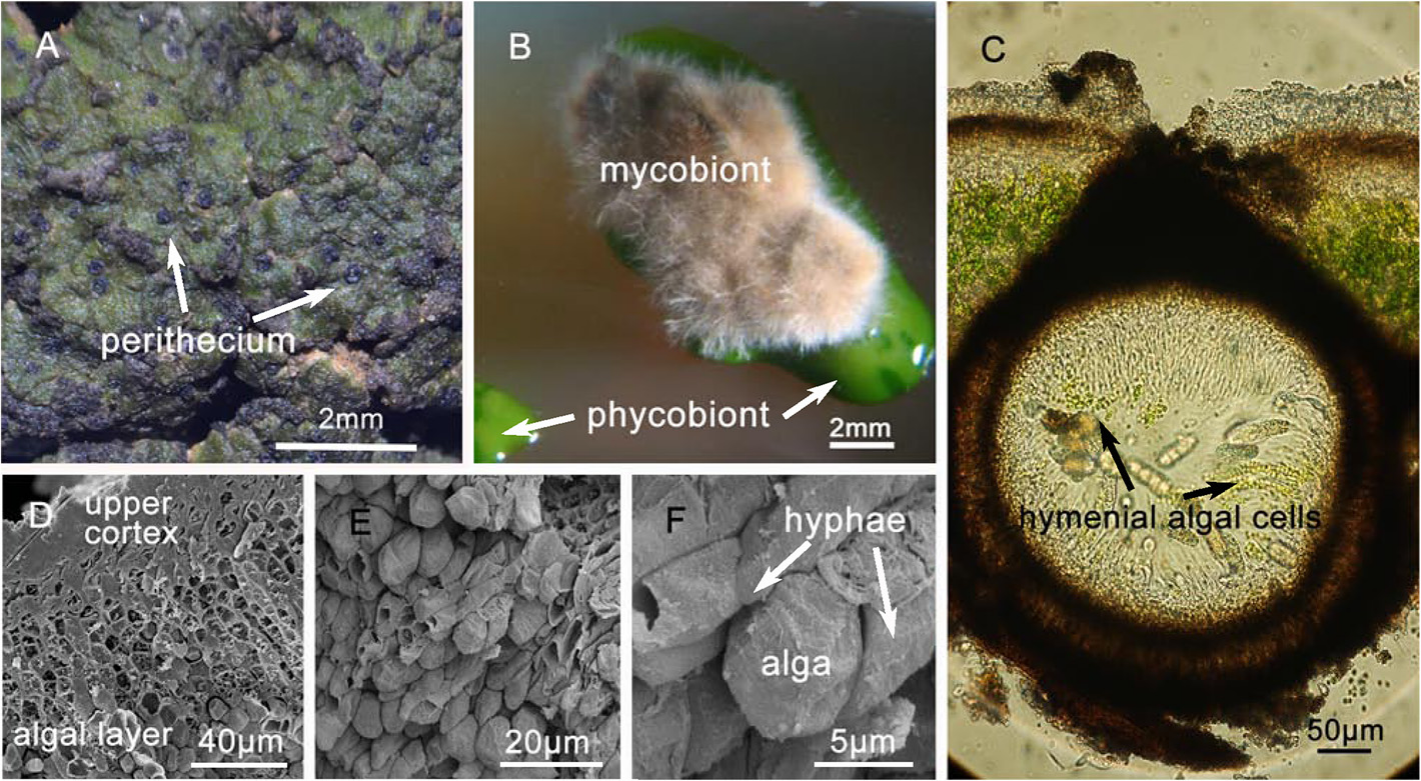
***Endocarpon pusillum*. A.** The lichen *E. pusillum*. **B.** The isolated mycobiont and phycobiont [26]. **C.** Cross section of a perithecium with hymenial algal cells inside. **D.** Cross section of a thallus under scanning electron microscopy (SEM). **E.** The algal layer (SEM). **F.** An algal cell is clasped and surrounded by some hyphae (SEM).

*Endocarpon* is a special genus, which have hymenial algal cells in their perithecia and the ascospores are discharged together with the hymenial algal cells [25]. The systematics and physiology of *E. pusillum* (Figure 1) have been studied well. The mycobiont *E. pusillum* exhibits much stronger desiccation-tolerant than other non-lichenized fungi as it can survive for 7 months under desiccation stress in combination with starvation stress [26]. Although a large number of fungal genomes have been published, no lichen-forming is included, and only two mitochondrial genomes from lichen-forming fungi (*Peltigera membranacea* and *Peltigera malacea*) and a transcriptome from the lichen *Cladonia rangiferina* have been reported until now [27,28]. Therefore, the genome of *E. pusillum* was sequenced and analyzed to ascertain the biological features of this lichen-forming fungus and the traits of lichenization.

## Results and discussion

### General features of the genome

**Figure 2:**
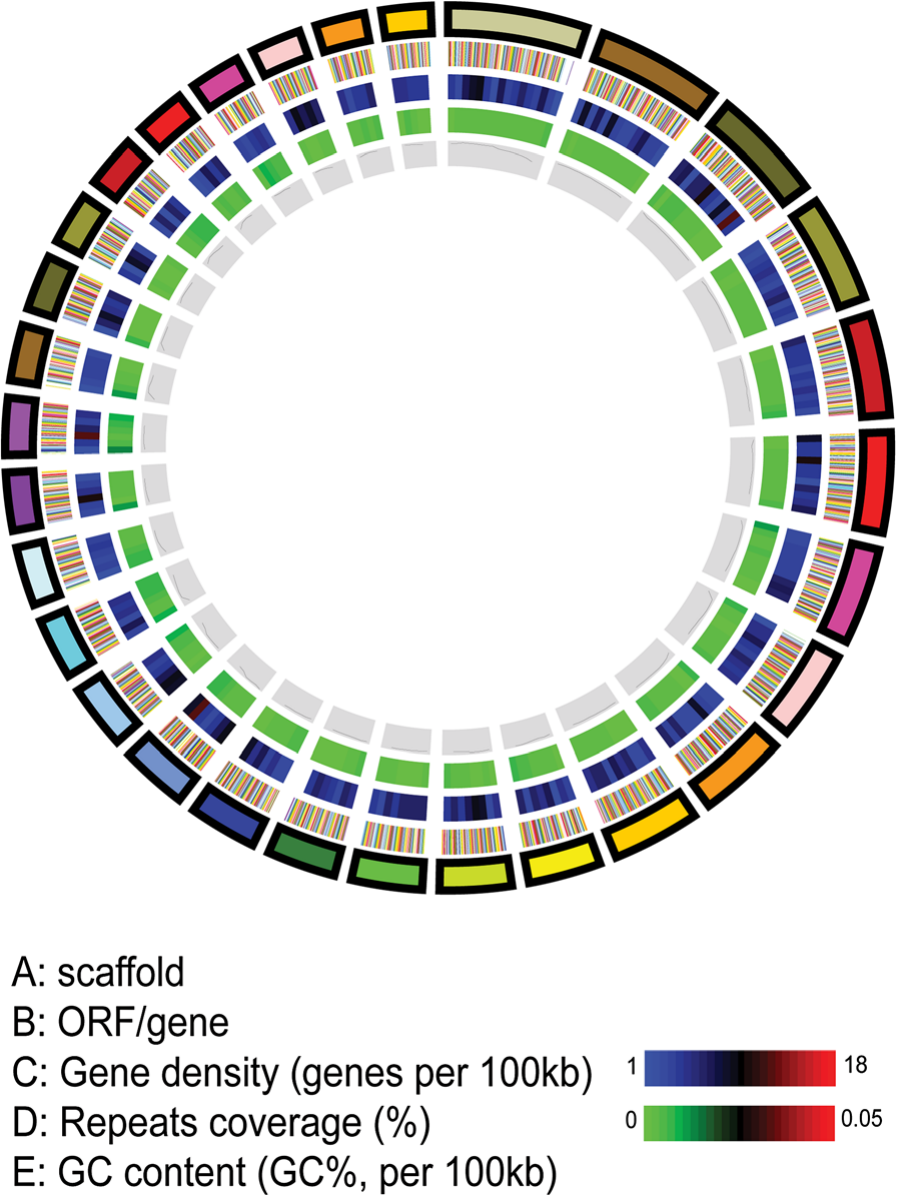
**Genome of *Endocarpon pusillum*.** A-E represent the circles from the outside to inside. **A:** Scaffolds of the genome, filtered by size (≥ 280 Kb). **B:** ORFs/genes. **C:** Gene density represented as number of genes per 100 kb (non-overlapping, window size 100 kb). **D:** Percentage coverage of repetitive sequences (non-overlapping windows, window size 100 kb). **E:** GC content was estimated by the percent GC in 100 kb non-overlapping windows.

The genome of the lichen-forming fungus *E. pusillum* was sequenced to about 78-fold coverage using both 454 and Illumina technologies (Additional file 1: Table S1). All sequences were assembled into 908 scaffolds (> 2 kb; N50, 178 kb) containing 1,731 contigs, with a genome size of 37.5 Mb (Table 1), which was almost identical to the result calculated by real-time polymerase chain reaction (PCR) [29]. The average GC content of the genome is 46.1%, and exonic region has a 4% higher GC content than the intronic region. Repetitive sequences represent 15% of the genome. A circular map was generated for the 30 largest scaffolds to illustrate the genome features more clearly (Figure 2).

**Table 1:** **Main features of the *Endocarpon pusillum *** **genome**

**Features**	***Endocarpon pusillum***
Assembly size/Mb	37.5
Scaffold N50/kb	178
Coverage/fold	78
G + C content	46.01%
GC Exonic	51.73%
GC Intronic	47.05%
Repeat rate	1.68%
Protein-coding genes	9,285
Gene density	250.8 per Mbp
Exons per genes	2.53
tRNAs	72
rRNAs	19
SM (Secondary Metabolism) genes	28
TE	15%

**Figure 3:**
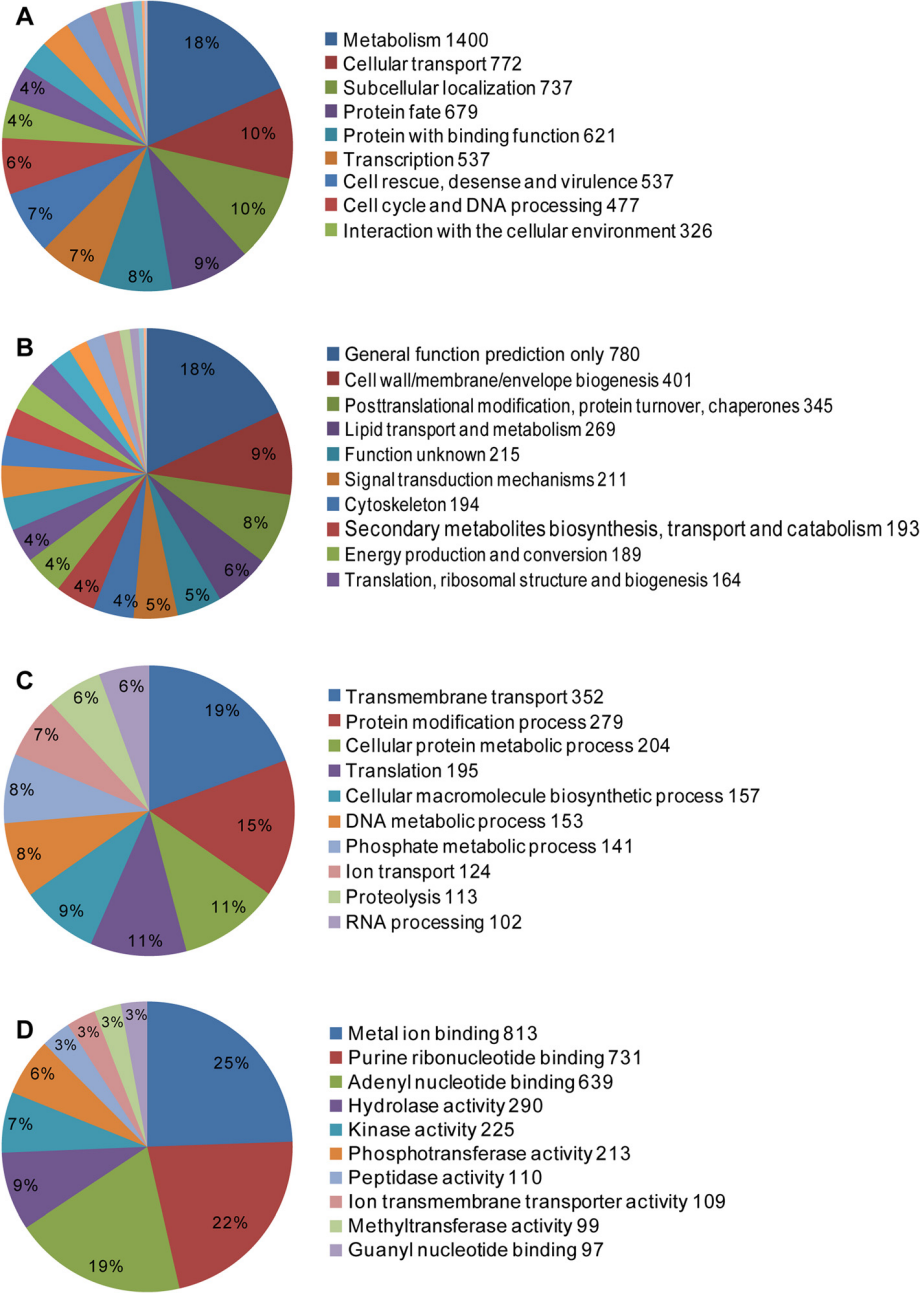
**Functional classification for the *Endocarpon pusillum *genome. A.** FunCat database classification in the second level. **B.** KOG database classification. **C.** The top 10 Gene Ontology biological process. **D.** The top 10 Gene Ontology molecular functions.

The whole project has been deposited at DDBJ/EMBL/GenBank under accession number APWS00000000. A total of 9,285 protein-coding genes were predicted, and 1,479 (15.7%) of these genes have no significant matches to known proteins from public databases. A total of 2,754, 3,787 and 7,589 proteins were assigned to Gene Ontology (GO) terms, the eukaryotic orthologous groups (KOG) and functional catalogue (FunCat) databases, respectively. The distributions of the top 10 GO, KOG, and FunCat terms of the sequences are presented in Figure 3.

### Phylogenetic analysis of *E. pusillum*

**Figure 4:**
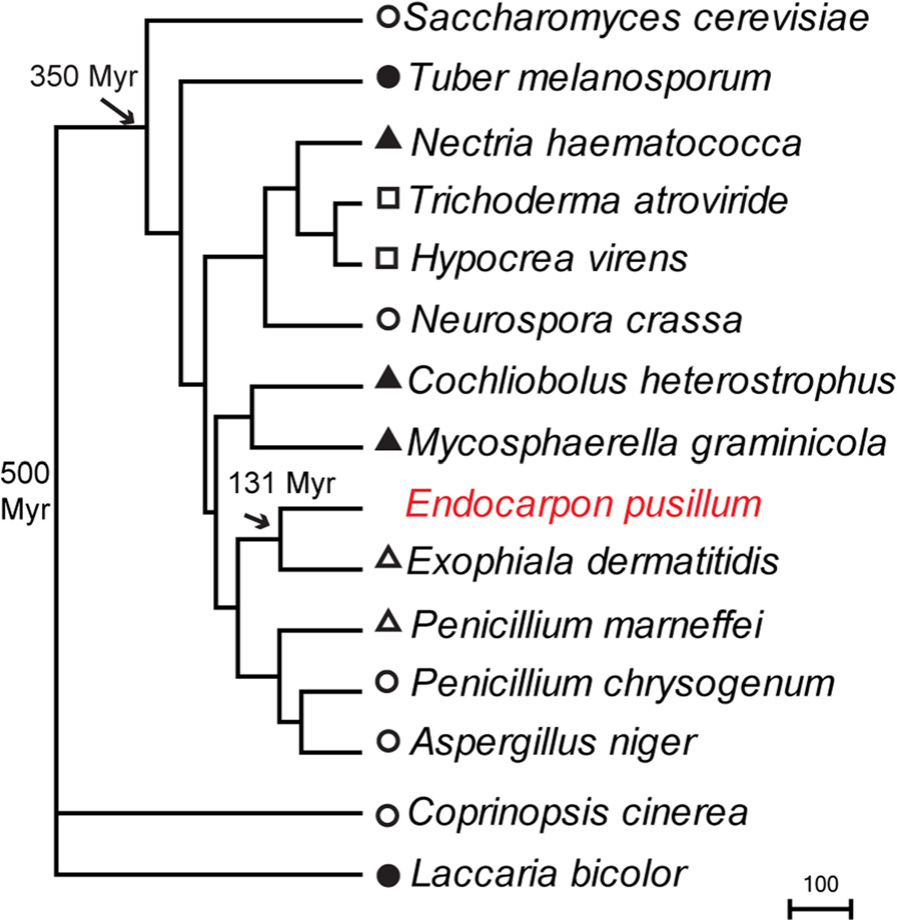
**Phylogenetic analysis of 15 fungi based on 1,893 orthologous genes.** The times of divergence of clades are indicated in millions of years by arrows. The symbols correspond to certain model fungi (open circles), mycorrhizal fungi (filled circles), plant pathogen (filled triangles), animal pathogen (open triangles) and fungi symbiosis with fungi (open squares). *C. cinerea* and *L. bicolor* belonging to the Basidiomycota were used as an outgroup.

The orthologous genes from the lichen-forming fungus *E. pusillum* and 14 other non lichen-forming fungi whose genomes were available were identified using Inparanoid [30] with default parameters (Figure 4). Phylogenetic analysis was performed using 1,893 single-copy orthologous genes identified among the genomes of above mentioned 15 fungi from the subkingdom Dikarya, and a linearized phylogenetic tree was constructed with estimates of the divergence times among these taxa (Figure 4). The phylogenomic analysis shows that the *E. pusillum* lineage is more closely related to the human pathogen *Exophiala dermatitidis*, which is the anamorph species of *Capronia* belonging to the Chaetothyriales, and the *E. pusillum* belongs to the Verrucariales. Both the orders belong to the same subclass Chaetothyriomycetidae, and the divergence between the lichenized Verrucariales and nonlichenized Chaetothyriales occurred approximately 131 million years (Myr) ago (Figure 4). This result is consistence with the phylogenetic analysis for Ascomycota using ribosomal RNA [9].

### Repeat-induced point mutation

Repeat-induced point mutation (RIP) is a gene silencing mechanism that can cause C-G to T-A mutations on repetitive DNA sequences, and the mutations from C to T mostly occur at CpA dinucleotides [31]. According to the method proposed by Margolin *et al.*[32], sequences with a high TA/AT ratio (>0.89) and a low (CA + TG)/(AC + GT) ratio (< 1.03) are thought to indicate RIP [33].

**Figure 5:**
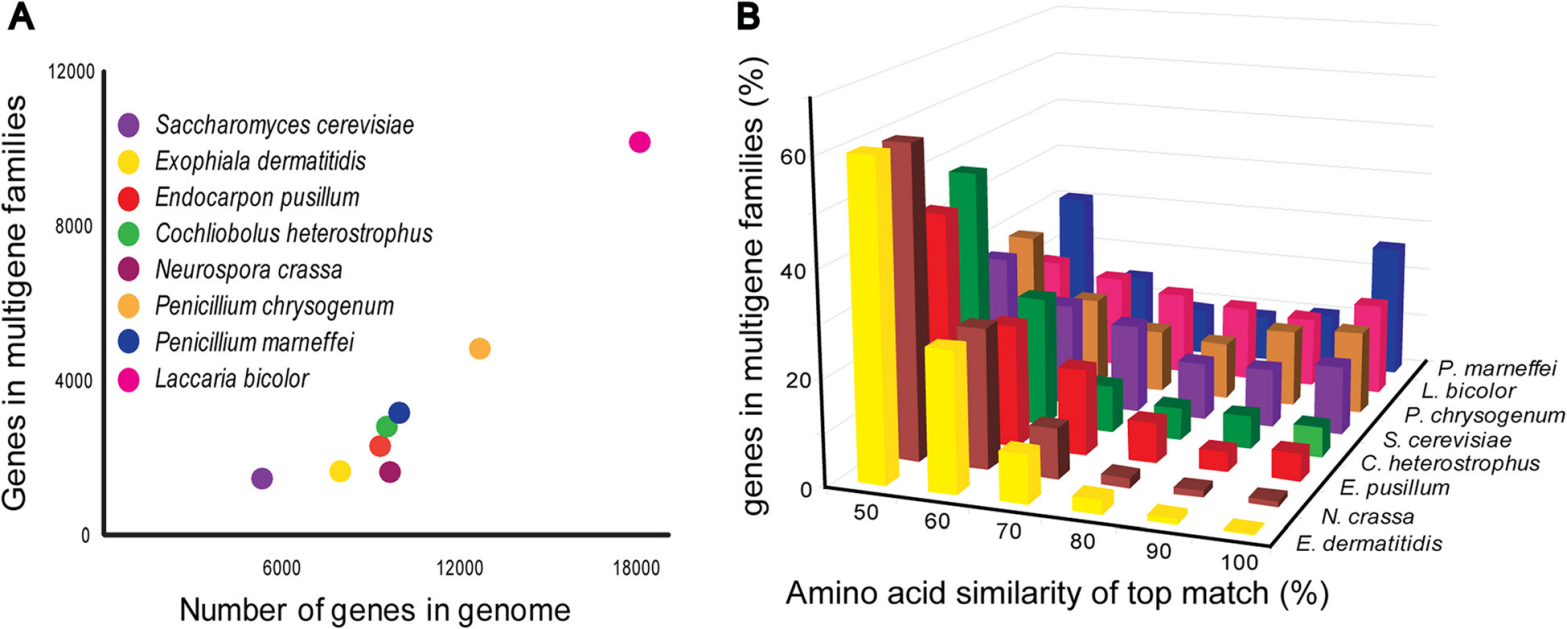
**Proportion of genes in multi-gene families. A.** The graph displays the proportion of genes in multigene families from *E. pusillum* and other fungi. **B.** Histogram of paralogous gene numbers with different levels of amino acid similarity in *E. pusillum* and other fungi.

A quantitative alignment-based method, RIPCAL [34], was used to search for evidence for RIP in *E. pusillum* genome. The RIP indices of the repetitive sequence are 1.46927 and 0.697168, and those of non-repetitive sequence are 0.565627 and 1.42277, which indicates that the regions of repetitive sequence in *E. pusillum* have undergone RIP. In addition, the percentage of genes in multigene families shows that *E. pusillum* has a low proportion of genes in multigene families (Figure 5A). The analysis of amino acid similarities in multigene families among eight fungi indicates that there are fewer best-matching (≥ 80%) genes in the *E. pusillum* genome (Figure 5B), which implies that some genes in the multigene families in *E. pusillum* are RIP mutated. However, the function of RIP in lichen-forming fungi still remains unclear.

### The mating systems and reproduction

**Figure 6:**
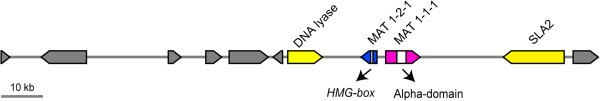
**Configuration of the *MAT *** **locus in *Endocarpon pusillum*.**

The genes involved in the mating process were identified in *E. pusillum* genome. The result shows that there is a single mating type (*MAT*) locus containing both a *MATα* and HMG domain in the genome, which provides the molecular evidence for homothallic lifestyle of *E. pusillum* (Figure 6). Genes for conidiophore development and asexual reproduction were also identified, together with those involved in fruit body development and sexual reproduction (Additional file 1: Table S2), and genes with the function of pheromone precursor (alpha-factor like), pheromone receptor (for alpha-factor like pheromone), ascus development (rhamnogalacturonase B), and receptors preventing improper sexual development have been lost in *E. pusillum*. However, it need more experimental evidence to determine whether these missing genes contribute to the special reproduction mode of *E. pusillum*.

### Secreted proteins

Secreted proteins are important effectors that modulate the interaction between pathogenic microbes and hosts [35-37], and many small secreted proteins (SSPs) are virulence factors [38]. However, *E. pusillum* has fewer secreted proteins than other phytopathogenic fungi (e.g. *Fusarium graminearum*, *Magnaporthe oryzae* and *Metarhizium anisopliae*) according to the blast results (Additional file 1: Table S3), and the function of 66% of SSPs is unknown. Lichen-forming fungus and pathogenic fungi are different, even though they both live with another organism and absorb nutrients from their partner. The analysis of the secreted proteins suggests that the SSP-mediated interaction between symbionts in *E. pusillum* is weaker than the virulence in parasitism (pathogenic fungi).

### Secondary metabolism

To date, several polyketide synthase (PKS) genes have been identified in different lichen-forming fungi [39-42], but no non-ribosomal peptide synthetase (NRPS) genes have been found. Analysis of the lichen-forming fungus *E. pusillum* genome revealed 2 NRPS genes and 14 PKS genes (Additional file 1: Table S4). The information of genes for secondary metabolism is helpful to the research of the secondary metabolites of *E. pusillum* and the synthesis conditions.

### Candidate genes for drought tolerance

At least 93 genes involving in these drought resistant mechanisms were identified in the genome of *E. pusillum* (Additional file 1: Table S5), which will provide us many important clues to resolve the question about why *E. pusillum* can survive in extreme drought environment. Recently, most studies on mechanisms about desiccation-tolerance in lichens have focused on the scavenging reactive oxygen species [43]. However, genes involved in regulating osmotic pressure, correcting protein misfolding, and scavenging the reactive oxygen species were identified in *E. pusillum* genome, which were worthy of further investigation.

### Multigene families expansion

Up to 1,889 protein families (containing at least two genes in all selected species) were identified from *E. pusillum* and 14 other fungi using Markov cluster (MCL) method. Based on BadiRate analysis, we found that 1,129 protein families were expanded and 760 protein families had undergone contraction in *E. pusillum*. The outlier results generated by BadiRate analysis are presented in Table 2. The expansion of these protein families might reflect specific characteristics of lichen-forming fungus *E. pusillum*.

**Table 2:** **Expanded and lineage-specific protein families in *Endocarpon pusillum***

**Protein family ID**	**Number of proteins in *E. pusillum***	**p-value**	**HMMPfam description**
group10696	20	0	Hypothetical protein CIMG_07368
group10024	15	0	Ankyrin and HET domain-containing protein
group14580	14	0	Ankyrin repeat
group10771	13	0	P-loop containing nucleoside triphosphate hydrolases
group11690	13	0	MFS general substrate transporter
group10071	11	0	Putative transposase
group10011	10	0	Hypothetical protein NECHADRAFT_87861
group14903	8	0	Heterokaryon incompatibility protein
group17019	7	1E-07	DNA/RNA polymerases
group10674	10	3E-07	Magnesium transport protein CorA, transmembrane region
group10674	10	3E-07	Hypothetical protein AN9301.2
group10038	8	5E-07	Hypothetical protein SS1G_06795
group10340	8	5E-07	Conserved hypothetical protein
group15154	6	5E-07	Hypothetical protein CHGG_10108
group16626	6	5E-07	Hypothetical protein
group18347	6	5E-07	Hypothetical protein
group10002	9	2.4E-06	Ribonuclease H-like
group10015	9	2.4E-06	Ankyrin repeat
group10097	7	4.6E-06	Ankyrin repeat
group10201	7	4.6E-06	ARM repeat
group10224	7	4.6E-06	Heterokaryon incompatibility protein
group10843	7	4.6E-06	Predicted protein
group11854	7	4.6E-06	Cytochrome P450
group10000	11	0.000001	P-loop containing nucleoside triphosphate hydrolases
group10705	5	0.000005	Hypothetical protein
group11683	5	0.000005	Dimeric alpha + beta barrel
group11909	5	0.000005	Unnamed protein product
group13886	5	0.000005	Ankyrin repeat
group15448	5	0.000005	FabD/lysophospholipase-like
group16624	5	0.000005	Protein kinase-like (PK-like)
group16962	5	0.000005	Hypothetical protein NFIA_004500
group17139	5	0.000005	Cytochrome P450
group18335	5	0.000005	Predicted protein
group18344	5	0.000005	Conserved hypothetical protein
group19016	5	0.000005	Hypothetical protein
group19022	5	0.000005	Hypothetical protein
group10704	8	1.78E-05	Conserved hypothetical protein
group10239	6	3.81E-05	P-loop containing nucleoside triphosphate hydrolases
group10495	6	3.81E-05	Unnamed protein product
group10677	6	3.81E-05	Unnamed protein product
group11482	6	3.81E-05	Predicted protein
group11606	6	3.81E-05	Hypothetical protein AOR_1_1448144
group15933	6	3.81E-05	Purine and uridine phosphorylases
group16361	6	3.81E-05	Hypothetical protein SS1G_13793
group16513	6	3.81E-05	Conserved hypothetical protein
group17376	6	3.81E-05	Hypothetical protein
group10014	9	4.84E-05	POZ domain
group10175	9	4.84E-05	Hypothetical protein CIMG_05613
group10238	4	4.94E-05	WD40 repeat-like
group11337	4	4.94E-05	TPR-like
group12140	4	4.94E-05	WD40 repeat-like
group16268	4	4.94E-05	Hypothetical protein MGYG_01629
group17166	4	4.94E-05	Predicted protein
group17167	4	4.94E-05	Alpha/beta-Hydrolases
group17374	4	4.94E-05	Hypothetical protein FRAAL5044
group17553	4	4.94E-05	Protein kinase-like (PK-like)
group17955	4	4.94E-05	Conserved hypothetical protein
group18350	4	4.94E-05	Hypothetical protein CHGG_08502
group19010	4	4.94E-05	NTF2-like
Lineage-specific protein families			
Protein family ID	Number of proteins in *E. pusillum*	p-value	Pfam description
group19669	4	4.94E-05	Ribonuclease H-like
group19844	4	4.94E-05	Transposase
group19847	4	4.94E-05	Hypothetical protein
group19848	4	4.94E-05	Hypothetical protein
group19850	4	4.94E-05	Protein kinase-like (PK-like)
group19853	4	4.94E-05	Ankyrin repeat

### Protein families involved in signal transduction

Compared with other fungi, the lichen-forming fungi are special because they can form mutualistic symbiosis with photosynthetic organisms and this specialty is reflected in its genome. Little is known about the functional genes in lichens, and most expanded protein families are function-unknown in *E. pusillum* (Table 2); however, it is likely that many of these expansion families are related to symbiosis. For example, the expressions of WD-repeat domain-containing proteins are up-regulated in the early developmental stages of lichen-symbiosis in *Cladonia grayi*[23].

Similarly, G protein-coupled receptors (GPCRs), which sense molecules outside the cell and activate signal transduction pathways inside the cell [44], are also likely to provide lichen-forming fungi with a strong ability to response to such signals, and all GPCR families have undergone obvious expansion (Table 3).

**Table 3:** **Evolutionary changes of GPCR protein families in *Endocarpon pusillum***

**GPCRs family**	**Number of protein in *E. pusillum***	**Evolutionary changes in *E. pusillum***	**Annotation**
Class A	6	Gain	Rhodopsin-like family
Class B	2	Gain	Secretin receptor
Class D	5	Gain	Fungal mating pheromone receptor
Class E	1	Gain	cAMP receptor

### Heterokaryon incompatibility protein families

There are 261 genes in the *E. pusillum* genome that were annotated to have heterokaryon incompatibility (HET)-related functions, and 182 of them are homologous to those in *N. crassa* and *Podospora anserina* (Additional file 1: Table S6). HI (Heterokaryon incompatibility) is a common characteristic among filamentous fungi; it can prevent the formation of heterokaryotic cells in which two different genomes coexist. Once the fusion between two individuals with incompatible *het* loci occurs, the HET genes would trigger the HI reaction, which is characterized as growth inhibition, repression of asexual sporulation, hyphal compartmentation and death of the heterokaryotic cell [45-47]. However, the biological significance of HI is still unknown. The expansion of HET protein families suggests that *E. pusillum* is likely to have a strict regulation of its vegetation [48], and may represent a strategy for stable genotype by defending against the transfer of exogenous genes.

### Lineage-specific protein families involved in self-splicing of insertions

The genes encoding ribonuclease H and transposase, which are predicted to be able to target some insertions, such as group I introns that can be stably integrated into the genome following the reverse splicing reaction [49], belong to lineage-specific multigene families in *E. pusillum* (Table 2).

The genomic sequencing data reveal that there are three group I introns in the large-subunit ribosomal DNA (LSU rDNA) of *E. pusillum* Z07020 at position 856, 2169, and 2721 (corresponding to the sequence of *Pichia methylivora* with NCBI accession number EU011611), and one group I intron in the small-subunit ribosomal DNA (SSU rDNA) at position 1769 (corresponding to the sequence of *Saccharomyces cerevisiae* with NCBI accession number Z75578).

Group I introns can self-splice themselves from an RNA transcript, requiring protein factors to facilitate the correct folding of the ribozyme core [49]. There are multiple group I introns present in the nuclear rDNA of many lichen-forming fungi [50-53], and they are considered to decrease the growth rate of lichen-forming fungi by interfering with rRNA maturation [54]. It is considered an adaption for lichenization: most lichens grow very slowly because the symbiosis would be disrupted if the mycobionts too many nutrients from the photobionts. Therefore, it is reasonable that the protein families involved in RNA reverse splicing have undergone expansion in a lichen-forming fungal genome that harbors abundant self-splicing introns.

### Protein families involved in transport

The exchange of ions and metabolites between the mycobiont and photobiont is the foundation of the symbiotic association. For this reason, genes encoding transporter proteins are expected to exhibit some traits in the lichen-forming fungus genome. There are 10 genes belonging to the magnesium transport protein CorA family, which showed significant expansion in the *E. pusillum* genome (Table 2). The CorA proteins were the first family to be identified that could transport Mg^2+^ in bacteria [55,56]. The mechanism of action of the CorA family has been well characterized in yeast and members of this family can transfer Mg^2+^ both into and out of the cell [57]. Magnesium is the most important divalent cation in cells, and is particularly important for photosynthesis [58]. Magnesium deficiency has been reported to affect plant photosynthesis and growth [59,60]. In the lichen thallus, the special structure of the symbionts (Figure 1D–F) makes it difficult for algal cells to absorb magnesium from the environment. However, genes for magnesium transport protein are expanded in *E. pusillum*, which led us to suspect that during symbiosis the mycobiont could provide magnesium to the phycobiont to meet the needs of their life cycles.

A striking finding is that most nitrogen transporter families are expanded but most sugar transporter families have been lost from the *E. pusillum* genome (Table 4). Though the mechanisms of substance transfer in lichen are not completely understood, it has been reported that the mycobiont absorbs certain carbohydrates generated by photosynthesis of the photobiont [61]. The reduction of sugar transporter genes, especially those for common sugars, such as glucose and fructose, which can be utilized by many organisms, suggests that the mycobiont simply absorbs certain uncommon carbohydrates from their photosynthetic partner. Therefore, the genes for many unnecessary sugar transporters have been lost during evolution. The lichen-forming fungi do not rely on common sugars because these carbon sources can be used by numerous microorganisms and lichen cannot compete with them for its slow growth and metabolism.

**Table 4:** **Evolutionary changes of transporter protein families in *Endocarpon pusillum *** **(partial results)**

**Transporter family**	**Number of protein in *E. pusillum***	**Evolutionary changes in *E. pusillum***	**Annotation**
1.A.11.3.3	3	Gain	Ammonium transporter-Hebeloma cylindrosporum
2.A.3.10.20	1	Gain	Lysine/arginine permease-*Candida albicans*
2.A.3.4.2	16	Gain	Gaba permease-Emericella nidulans-*Aspergillus nidulans*
2.A.3.8.4	6	Gain	High affinity methionine permease-*Saccharomyces cerevisiae*
2.A.39.3.3	1	Gain	Uridine permease-Saccharomyces cerevisiae
8.A.9.1.1	3	Gain	Amino acid transport related protein (RBAT)-*Oryctolagus cuniculus*
2.A.1.1.10	0	Loss	Maltose permease MAL6T-*Saccharomyces cerevisiae*
2.A.1.1.11	1	Loss	General alpha-glucoside permease-*Saccharomyces cerevisiae*
2.A.1.1.33	0	Loss	Hexose transporter-*Kluyveromyces lactis*
2.A.1.1.38	2	Loss	Sugar transporter STL1-*Saccharomyces cerevisiae*
2.A.1.1.39	3	Loss	High-affinity glucose transporter-*Kluyveromyces lactis*
2.A.1.1.57	1	Loss	Monosaccharide transporter-*Aspergillus niger*
2.A.1.1.58	0	Loss	Monosaccharide transporter-*Aspergillus niger*
2.A.1.1.68	0	Loss	Glucose transporter/Sensor OS-*Pichia stipitis*
2.A.1.1.9	0	Loss	Lactose permease-Kluyveromyces lactis
2.A.1.2.23	1	Loss	Fructose facilitator-Zygosaccharomyces bailii

By contrast, the expansion of nitrogen transporters suggests that the lichen-forming fungi need to transfer (export or import) various nitrogen sources. Nitrogen is an indispensable substance for the growth of organisms, and lichens whose photosynthetic partners are green algae must obtain inorganic and organic nitrogen [62-64]. There is no evidence for nitrogen transfer between symbionts in lichen. However, owing to the particular structure of lichen thallus in which the photobiont cells are tightly entwined by mycelia (Figure 1D–F) and the photobiont cells cannot absorb substances from their surroundings directly, it can be deduced that the necessary components for their growth, such as ions and nitrogen sources, must be transferred from the mycobiont to the photobiont. The phycobiont may be have a preference for certain forms of nitrogen; for example, in the alga-bacteria association, the algae prefer NH_4_^+^ and NO_3_^-^, which are release by bacteria when cultured in organic nitrogen conditions [65]. Thus, the mycobiont may absorb and transform the nitrogen forms that are preferred by its photosynthetic partner. Therefore, the expansion of nitrogen transporters should be related to this physiological process. Recently, it was reported that the ammonium transporter, MEP-α, in lichen-forming fungi was obtained though horizontal gene transfer [66], which can partly explain the mechanism of the expansion of the nitrogen transporter superfamily. This gene appears to have been lost in some groups of lichens, such as in those that are symbiotic with nitrogen-fixing cyanobacteria or those that inhabit high-nitrogen niches [67]. However, for the lichens whose photobiont is a green alga that cannot fix nitrogen, the expansion of nitrogen transporter families is of great significance in promoting the growth of the lichen thallus, as seen in *E. pusillum*.

### Expression analysis of symbiosis-related genes by quantitative real-time PCR (qRT-PCR)

A qRT-PCR experiment was performed to determine the changes of some symbiosis-related genes in the lichen-forming fungus at transcription level when *E. pusillum* pre-contacted with its photosynthetic partner, *Diplosphaera chodatii*.

### Symbiosis-related genes selection

Thirty-two genes encoding lectins were identified in the *E. pusillum* genome. They belong to four superfamilies: concanavalin A-like lectin, fucose-specific lectin, mannose-binding lectin and ricin B-related lectin. For the qRT-PCR assay, six lectin genes were selected as representatives of the four superfamilies, according to the difference in the number of transmembrane helices and signal peptides (Additional file 1: Table S7).

The expansions of nitrogen transporters and the contractions of sugar transporters imply that they are closely related to maintenance of both symbionts. Hence, certain genes encoding these transporters were chosen for the qRT-PCR assay. Among them, two ammonium transporters and one nitrate transporter that displays high homology to those in other fungi (Additional file 1: Table S8) were included. It has been reported that eight sugar transporters are able to transfer photosynthesis products in other fungi, and they are predicted to be functional at the interface of lichen association [68-75]. Thus, the homologous proteins were identified in the *E. pusillum* genome (Additional file 1: Table S9). The genes sharing homology with known transporters were obtained using BlastP and each of them appeared in several BlastP results; their expressions were determined by the qRT-PCR assay (Additional file 1: Table S10). Nitrogen metabolism and sugar metabolism in fungal cells would be active after nitrogen and carbohydrates were assimilated or transferred. Therefore, genes encoding glutamate synthase and nitrite reductase (Additional file 1: Table S8), and that encoding the Golgi GDP-mannose transporter, which is functional in the glycosylation of secreted proteins [76] and five enzymes involved in galactose and nucleotide sugar metabolism (Additional file 1: Table S10) were also included. The gene encoding the tetracycline resistance protein was chosen as a control, because it recognizes and exports tetracycline from the cell [77,78] and is expected to show no expression change under the experimental conditions in the present study.

### Differences in expression levels of genes involved in symbiosis under co-culture conditions

**Figure 7:**
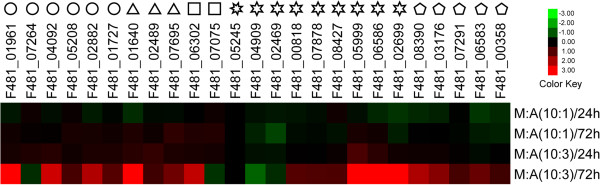
**Relative transcription levels of certain symbiosis-related genes in *Endocarpon pusillum *when cultured with *Diplosphaera chodatii*.** The names of the genes are listed on the top of the picture. Circles represent genes encoding lectins, triangles represent N transporter genes, squares represent gene encoding enzymes of nitrogen metabolism, a heptagon represents the tetracycline resistance protein gene, hexagons represent sugar transporter genes, and pentagons represent genes encoding enzymes of sugar metabolism. The culture conditions are displayed at the right of the picture, in which the ratios of mycobiont (M) and phycobiont (A) and incubation time are given. The log_2_ relative expression with respect to the control group is illustrated in the heat map.

From the results presented in Figure 7, the expression levels of most genes increased only under the condition for experimental group IV (Table 5) (weight ratio of lichen-forming fungus and phycobiont is 10:3, and culture time is 72 hours), which indicated that the contact time and biomass ratio of both symbionts affects their recognition and nutrient transfer significantly.

**Table 5:** Sample treatments to identify differentially expressed genes

**Experiment group**	**Sample**	**Weight ratio (mycobiont:Phycobiont)**	**Incubation time (h)**
Control A	Mycobiont	-	24
Control B	Mycobiont	-	72
Control C	Phycobiont	-	72
I	Mycobiont + phycobiont	10:1	24
II	Mycobiont + phycobiont	10:3	24
III	Mycobiont + phycobiont	10:1	72
IV	Mycobiont + phycobiont	10:3	72

Three lectin genes (F481_01961, F481_04092, F481_02882) showed significant increases (log_2_ fold change > 2) in their expression level in experiment group IV compared with the control group B (only the mycobiont was cultured in BBM for 72 h) (Table 5; Figure 7). Interestingly, the common aspect of these lectins is that there are no predicted signal peptides in their amino acid sequences (Additional file 1: Table S7) (http://elm.eu.org) [79]. Lectins are sugar-binding proteins that play an important roles in cell recognition [80]. The first lectin discovered in lichens was that found in *Peltigera canina* and *Peltigera polydactyla*[17]. Thereafter, some studies showed that lectins secreted by lichen-forming fungi may be involved in recognizing their compatible algae [15,16,81]. Lectins, as surface proteins, can directly contact compatible phycobionts, and the contact requires receptor sites on the surface of the phycobiont to which the lectins binds [82,83]. Recent studies on lectins in *P. membranacea* indicated that the expression of galectin *lec-1* was influenced by the presence of the phycobiont [84], and the evolution of galectin *lec-2* was driven by interaction with different strains of the phycobiont *Nostoc*[85]. Galectins, together with cellular slime mold lectin discoidin I, do not have signal peptides in eukaryotic cells and both of them have intracellular and extracellular localizations [86,87]. Our study showed that lectins without signal peptides could play a major role in symbiotic recognition and provided a practical guide for screening for lectins participating in the interaction between lichen-forming fungi and their photosynthetic partners.

An ammonium transporter (F481_01640) and a nitrate transporter (F481_07695) were up-regulated (log_2_ fold change > 2) in experimental group IV (Figure 7). BBM contains nitrate ions; therefore, the up-regulation of the nitrate transporter (F481_07695) is expected. However, the up-regulation of the ammonium transporter (F481_01640) suggests that ammonium could be produced from nitrate in *E. pusillum* and transferred to its photosynthetic partner, because there is no ammonium ion in BBM. The differences in the expressions of glutamate synthase (F481_06302) and nitrite reductase (F481_07075) indicate that nitrogen metabolism is altered in the lichen-forming fungus when it meets its compatible alga. It has been reported that algae from different lichens can utilize organic and inorganic nitrogen [62-64]. Thus, we can conclude that the pattern of nitrogen transfer in *E. pusillum* is that the mycobiont absorbs various nitrogen sources from the environment and can convert them to different forms *in vivo* to meet the algae’s demand for nitrogen, based on the expansion of nitrogen transporter families in *E. pusillum* genome and the result of co-culture experiments.

The lichen-forming fungus cannot obtain any organic nutrition from BBM. However, six of the symbiosis-related sugar transporter genes (F481_00818, F481_07878, F481_08427, F481_05999, F481_06586, and F481_02699) show significant differences in expression in experiment group IV (Figure 7). This suggests that some carbohydrates are released into the BBM by *D. chodatii*, and the transporters encoded by these genes may be responsible for transferring these carbohydrates into the fungal cells. The upregulation of the expression of the Golgi GDP-mannose transporter (F481_08390) under experimental group IV conditions, bearing in mind that a previous study proved that this transporter participates in glycosylation [76], indicated that the sugar metabolic pathway is active in *E. pusillum* after obtaining carbohydrates. Furthermore, the enzymes (F481_03176, F481_07291, F481_06583, and F481_00358) involved in galactose and nucleotide sugar metabolism also showed high expressions levels, which demonstrated that the carbohydrates produced by the alga and absorbed by the fungus are indeed used for fungal cell life activities.

### Native and non-native sugars utilized by *E. pusillum*

The carbohydrates used for growth and metabolism of lichen-forming fungus originate from the photosynthetic products of its photosynthetic partner, however, the forms of the photosynthetic products absorbed by different lichen-forming fungi vary depending on their different photobionts. It has been report that lichen-forming fungi absorb many polyols or glucose from algae or cyanobacteria, respectively [20,21], and the photosynthetic product transferred from D. chodatii to E. pusillum was sorbitol [20]. Additionally, previous studies showed that some monosaccharides, such as glucose, and disaccharides, such as trehalose and sucrose, were transferred between non-lichenized symbionts [68-75]. Because some homology structures are frequently found in different sugar transporters from diverse fungi, the specificities of these transporters may be low. Therefore, 11 different carbohydrates were used to confirm the function of sugar transporters in E. pusillum and the potential carbohydrates transferred into this fungus.

**Figure 8:**
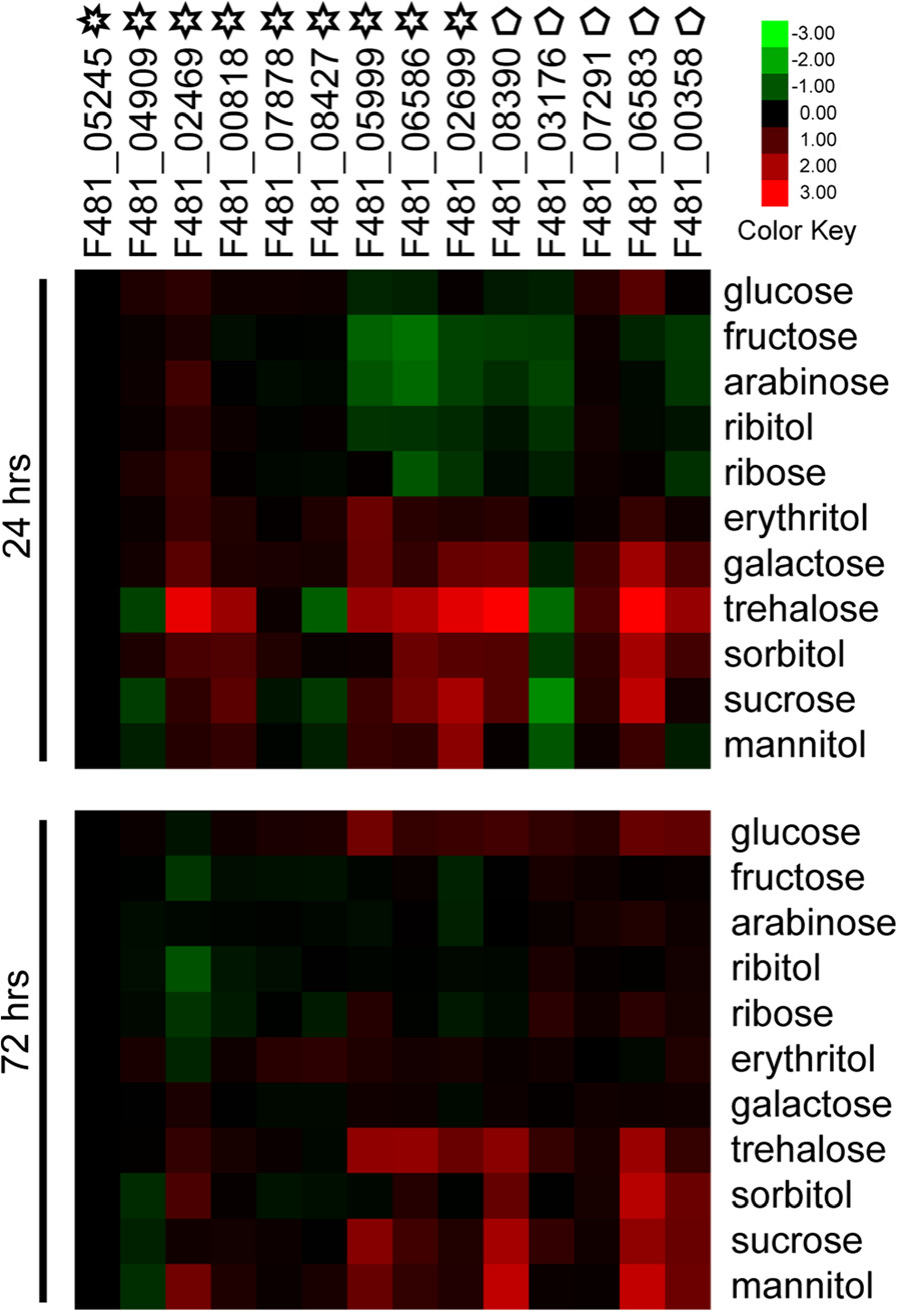
**Relative transcription levels of sugar transfer-related genes in *Endocarpon pusillum *after supplementation with different carbohydrates.** The names of the genes are listed on the top of the picture. Heptagons represent the gene encoding the tetracycline transporter, hexagons represent sugar transporter genes, and pentagons represent genes encoding enzymes of sugar metabolism. The culture time is displayed on the left and sugars added into BBM are listed on the right. The log_2_ relative expression with respect to the control group is illustrated in the heat map.

The most significant up-regulation in expression of the examined genes was in the sample with trehalose for 24 hours. However, the high expression levels were not maintained over the next 48 hours (Figure 8). This suggests that trehalose cannot be absorbed by the lichen-forming fungus as an energy source, but is likely to be a signal molecule for the activation of the gene expression in E. pusillum. Trehalose is involved in many functions besides osmoprotection [88], and acts as a signal molecule that is possibly exported from bacteria to plants to regulate the carbon and nitrogen metabolism of plants in plant-bacteria interactions [89]. Trehalose can be converted into trehalose-6-phosphate (T6P), which inhibits hexokinase activity and regulates glycolysis. Over expression of the trehalose 6-phospate synthase (TPS) gene induces the expression of several genes involved in stress tolerance, and carbon and nitrogen metabolism [90,91]. In the alga-invertebrate association, trehalose produced by the alga was shown to be present in certain symbiotic interfaces [92]. Although no research has shown a link between trehalose and lichen symbiosis interaction, our results suggest that trehalose may act as a signal molecule and would play an important role in the symbiotic interaction in E. pusillum.

Four carbohydrates (glucose, sucrose, sorbitol, and mannitol) increased the transcription levels of sugar transporters or enzymes involved in sugar metabolism in *E. pusillum* at 24 and 72 hours (Figure 8). These results suggest that these carbohydrates maintain the metabolism of the lichen-forming fungus over a long period, which implies that *E. pusillum* may absorb them as its carbon sources through some common sugar transporters. Although the transcription levels of some genes slightly fluctuated at 24 and 72 hours in the presence other sugars (arabinose, erythritol, fructose, galactose, ribitol and ribose) in the BBM, no genes exhibited a clear rising trend compared with the control group, especially those involved in sugar metabolism (Figure 8). This indicates that these sugars cannot be utilized as carbon sources by *E. pusillum*.

The transcription levels of some sugar transporters, such as F481_05999, F481_06586, and F481_2699, were up-regulated when various sugars were added to the BBM, indicating that they can transfer more than one carbohydrate into *E. pusillum*. Some genes showed different changes in their transcription levels, either in trend or extent, compared with those in the co-culture experiment. For example, sugar transporter F481_02469 showed no differential expression in the co-culture experiment, but was up-regulated slightly under some sugars treatments. In addition, the transcription levels of sugar transporters F481_05999, F481_06586, and F481_02699 were up-regulated more obviously in the co-culture experiment (Figures 7 and 8). These phenomena imply that there is some induction mechanism between the symbionts; i.e., the expressions of certain genes in a lichen-forming fungus are influenced by its photosynthetic partner to absorb organic carbon effectively.

**Figure 9:**
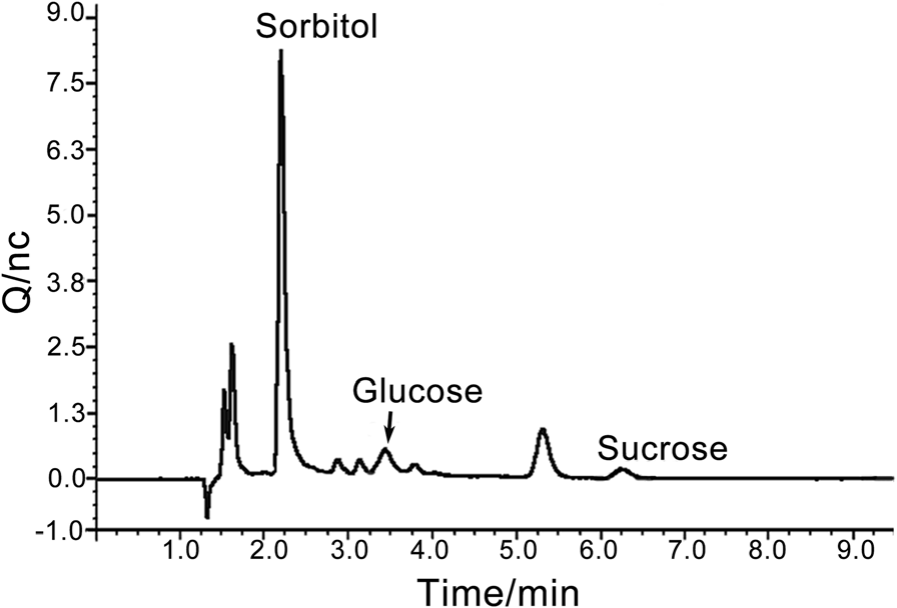
**Ion chromatogram of co-cultured sample from experimental group IV.** The sample from experiment group IV (mycobiont: phycobiont = 10:3; culture for 72 hrs) was diluted 25 times before four carbohydrates (glucose, mannitol, sorbitol and sucrose) were measured using ion chromatography.

Ion chromatography was used to detect glucose, sucrose, sorbitol and mannitol in the filtrates of experiment group IV, and controls B and C, to determine whether they could be released by *D. chodatii* and to determine the conditions under which this alga secretes organic carbon into the BBM. Sorbitol (12.6 mg/100 mL), glucose (0.8 mg/100 mL) and sucrose (0.8 mg/100 mL) have been detected in group IV (Figure 9). Thus, sorbitol showed the highest accumulation; however, there were other, unidentified, carbohydrates, which suggested that the lichen-forming fungus *E. pusillum* preferentially absorbs sorbitol. By contrast, no mannitol was found in this sample. There were no detectable carbohydrates in the control group B and C (the phycobiont and the lichen-forming fungus cultured respectively in BBM), which demonstrated that the pure cultured *D. chodatii* does not release carbon. Previous experiments for the phycobionts of *Ramalina crassa* and *Ramalina subbreviuscula* indicated that the pure cultured phycobiont released ribitol to the medium [19]. However, Hawksworth (1984) suggested that the photobiont had lost its ability to release carbohydrate after isolation from the lichen thallus, and that the lichen-forming fungus exerted some specific control on the photobiont cells that lead them to secrete carbohydrates [21]. Our result implies that *E. pusillum* can control the sugar export of *D. chodatii*, and that the biomass ratio and contact time are crucial for the interaction between the two symbionts.

Although mannitol is not released by D. chodatii, it can still be utilized by E. pusillum; it is likely to be because mannitol and sorbitol are isomers. This suggests that E. pusillum has the potential to use non-natural carbon sources. This phenomenon is likely to occur in other lichen-forming fungi, which would contribute to discovering the mechanism of the algal switch [93-95] and could provide clues for the evolution of lichenization.

## Conclusions

Approximately 40% of all Ascomycota are lichen-forming; thus, lichenization is regarded as one of the most important fungal lifestyles [96]. Hence, genomic information for lichen-forming fungi would expand the knowledge of fungi. In the present study, we report, for the first time, the characteristic of the lichen-forming fungal genome, which displays many features that are different to other fungi.

This is the first study to report that the lichen-forming fungal genome have undergone RIP. Genes for mating system, secondary metabolism, and the drought-related mechanisms were indentified in *E. pusillum* genome, which are worth being investigated in the future. The evolution analysis of multigene families indicated the expansion and contraction in *E. pusillum* genome reveal the effect of lichenization on lichen-forming fungi.

Co-culture experiments suggest that the lectins without signal peptides would be likely to play an essential role in the recognition of lichen symbiosis, and one of the most striking findings in these experiments is that an appropriate weigh ratio of lichen-forming fungus and its photosynthetic partner and sufficient contact time are vital for their recognition and mutual influence. We also confirmed that the most important natural carbon source for *E. pusillum* is sorbitol transferred from *D. chodatii*; however, this lichen-forming fungus can also use other non-natural carbohydrates under the pure culture condition.

**Figure 10:**
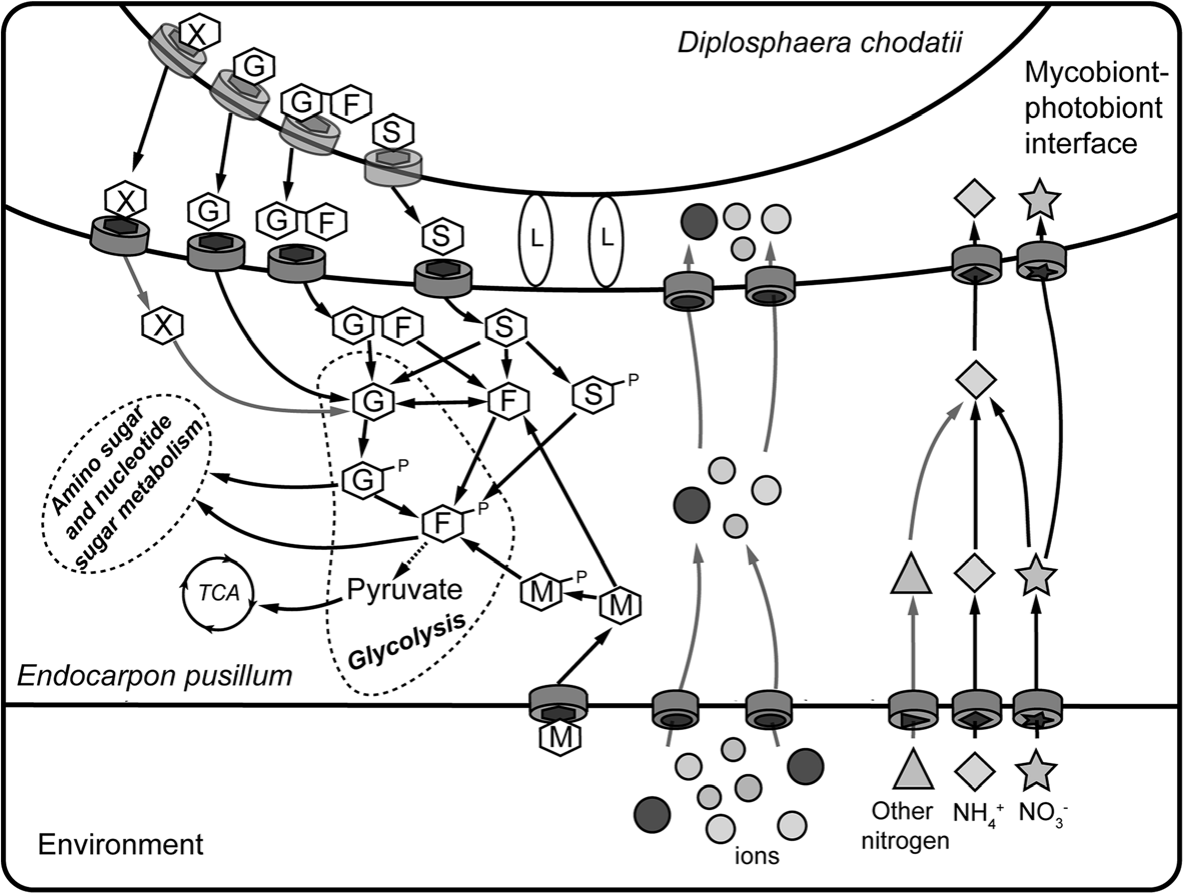
**Summary of the interactions between symbionts in lichen *Endocarpon pusillum*.** The lectins lacking signal peptides, which are shown as ellipse marked with L, act as recognition factors in the direct contact between *E. pusillum* and *D. chodatii*. After it is captured by the compatible fungal partner, the photosynthetic products sorbitol (S), glucose (G) and sucrose (G-F) are released from the phycobiont *D. chodatii.* These sugars are then absorbed through sugar transporters of *E. pusillum* and converted into glucose or fructose to support fungal metabolism. In addition, there are a small amount of other uncertainty carbon sources (X) released by the phycobiont, and the mycobiont can also utilize mannitol (M) from environment. At the same time, organic and inorganic nitrogen, together with various ions in the environment, are transferred into *E. pusillum*. In fungal cells, these substances may be converted into the forms preferred by the phycobiont and are then delivered to *D. chodatii*.

A mycobiont-phycobiont interplay model is shown as Figure 10. The model reflects aspects of the recognition and interaction of the lichen thallus in *E. pusillum* and is likely to be applicable to other lichens, especially those whose photobionts are algae. This study provides a valuable genomic resource for future research in screening functional genes including drought-tolerance genes from lichens and would be useful for investigating the formation and divergence on the functional biology between lichenized and nonlichenized fungi.

## Methods

### Fungal strains

The lichen-forming fungal strain of *E. pusillum* Z07020 (HMAS-L-300199) was isolated by a single-spore discharge from the perithecium of lichen *E. pusillum* collected from Shapotou Desert Research Station (SDRS) of the Chinese Academy of Sciences (CAS) in the Tengger Desert of northern China [26]. The isolates were grown on 1.5% water agar for 1–2 weeks, and then cultured at room temperature after transfer to potato dextrose liquid medium.

### Genome sequencing and assembly

The genome of the lichen-forming fungus E. pusillum was sequenced using high-throughput next-generation sequencing technology and the sequencing platforms were Roche 454 and Illumina Solexa systems. Genomic libraries containing 8-kb inserts were constructed and 1,394,086 paired-end reads (281.9 Mb) were generated using the 454 Roche GS FLX system. The Illumina adaptors were ligated onto the genomic DNA fragments, and DNA fragments with estimated sizes of 0.5 kb to 3 kb were selected using gel-electrophoresis. Libraries were PCR-amplified using Phusion polymerase. Sequencing libraries were denatured with sodium hydroxide and diluted in hybridization buffer for loading onto a single lane of an Illumina GA flow cell. A Solexa sequencer generated the mate-paired reads (7,155,072 reads, 716 Mb) and paired-end reads (18,176,986 reads, 1818 Mb). Solexa sequencing paired-end reads and mate-paired reads were assembled by SOAPdenovo [97], which adopts the de Bruijn graph data structure to construct contigs.

### Gene prediction and annotation

We used Augustus [98], GeneID [99], and GeneMark-ES [100] programs to predict the gene models for E. pusillum. A final set of gene models was selected by EvidenceModeler [101]. An ab initio prediction was carried on using the annotated information of A. fumigatus as a reference. All predicted gene models were subjected to GO [102], KOG [103], FunCat [104] and Kyoto Encyclopedia of Genes and Genomes (KEGG) database analysis [105]. Protein domains were predicted using InterProScan [106] against various domain libraries (HMMPfam, superfamily, HMMTigr, and HMMSmart). Repetitive elements were identified by blasting against the RepeatMasker library (http://www.repeatmasker.org/). Non-coding RNAs were predicted according to the Rfam database [107], and tRNAs were predicted using tRNAscan-SE [108]. Pseudogenes and rRNAs were designated using PseudoGene [109] and RNAmmer [110] respectively.

### Orthologous gene and phylogeny analysis of *E. pusillum*

The sequences of corresponding orthologous genes from 15 fungi were aligned using ClustalW [111]. A maximum parsimony (MP) phylogenomic tree was created using the concatenated amino acid sequences in phylogenetic analysis using parsimony (PAUP) [112], and a bootstrap analysis with 1000 replications was performed to evaluate the reliability of the phylogenetic tree. The divergence time between species was estimated using the r8s method [113]. The time of divergence between Ascomycota and Basidiomycota is set as 500 Myr, and the time between Pezizomycotina and Saccharomycotina was 350 Myr [114].

### Multigene families and evolution analysis

The families that were absent from the most recent common ancestor were chosen to analyze the evolution of protein families [115]. Multigene families were identified using the MCL method [116]. Whole genome blast analyses against Transporter Classification Database (http://www.tcdb.org/tcdb/) and GPCRDB (http://www.gpcr.org/7tm/) database were performed to identify genes exhibiting difference between E. pusillum and other fungi. All proteins in the genome were blast searched against the database with an e-value cutoff of ≤ e^-10^ and at least 40% identity over 60% coverage. A new software, BadiRate [117], was used in the evolutionary analysis of multigene families to estimate rates of gene gain and loss. Families with p-values less than 0.01 were considered to have experienced significant expansion or contraction.

### Mating type

To detect the sexual cycle of E. pusillum, genes involved in the mating process, incompatibility, ascomata and conidiophore development, and HET were identified using BlastP against related genes from A. niger, A. nidulans [118], N. crassa [119] and P. anserine [120,121].

### Analysis of genes involved in secondary metabolism

Genes encoding PKS, NRPS, and NRPS-PKS hybrid genes in the genome of E. pusillum were analyzed with the program SMURF (http://www.jcvi.org/smurf/index.php). Modulation analysis and domain extraction of different NRPS or PKS proteins were conducted by Blast searching against the SBSPKS database [122].

### Secreted proteins

The potential secreted proteins of *E. pusillum* and other fungi, including *F. graminearum*, *M. oryzae*, and *M. anisopliae*, were predicted by SignaIP 3.0 analysis using a hidden Markov model [http://www.cbs.dtu.dk/services/SignalP/].

### Quantitative RT-PCR

RNA was extracted from cultured lichen-forming fungus *E. pusillum*. cDNA synthesis and relative quantitative RT-PCR were carried out as described previously [28]. For each treatment, qRT-PCR was performed in an Applied Biosystems 7500 Real-Time PCR system (Applied Biosystems, USA). The data were analyzed using the 2^-ΔCt^ method.

### Gene-stability measure of reference genes using geNORM

Vandesomepele et al. (2002) developed an algorithm named geNORM that determines the expression stability of reference genes [123]. The calculated gene-stability measure (M) relies on the principle that the expression ratio of two ideal internal reference genes is identical in all samples, regardless of the experimental condition or cell type. Ten genes whose expressions showed no difference in comparative transcriptome data between control and drought-stress conditions (unpublished data) were chosen to determine the best reference genes (Additional file 1: Table S11). Ultimately, we chose the tetracycline resistance protein (F481_ 05245) gene as the reference gene.

### Treatment of samples for co-culture experiments

Four treatments were carried out, comprising two weight ratios (10:1 and 10:3) for the lichen-forming fungus and phycobiont and two culture times (24 h and 72 h) (Table 5), to investigate whether the ratio and contact time between the lichen-forming fungus and phycobiont has an effect on gene expression in *E. pusillum* when both symbionts are incubated together.

The experiment was performed in Bold’s basal medium (BBM) (does not contain carbohydrate), and under 12 h illuminations per 24 h, which allow the algal cells to produce carbohydrates by photosynthesis.

### Treatment of samples for sugar transfer experiments

To confirm the capability of *E. pusillum* to utilize carbohydrates, polyols (mannitol, sorbitol, ribitol, and erythritol), monosaccharides (glucose, fructose, arabinose, ribose, and galactose) and disaccharides (trehalose and sucrose), respectively, were added to BBM containing only the lichen-forming fungus, and the transcript levels of genes involved in sugar transport and metabolism were determined after culturing for 24 and 72 hrs.

### Determination of photosynthetic products by ion chromatography

Ion chromatography was used to determine glucose, sucrose, sorbitol and mannitol in co-cultured samples. The analytes were separated on a CarboPac™ PA1 (4 mm × 250 mm) anion exchange column using 200 mmol/L NaOH as mobile phase at flow rate of 1.0 mL/min and detected with a pulsed amperometric detector.

## Availability of supporting data

The data sets supporting the results of this article are included within the article (and its additional files).

## Abbreviations

BBM: Bold’s basal medium; bp: Base pair; FunCat: Functionally annotation; GO: Gene ontology; GPCR: G-protein coupled receptor; HET: Heterokaryon incompatibility; HI: Heterokaryon incompatibility; HMAS: Mycological herbarium of microbiology institute,the chinese academy of sciences; KEGG: Kyoto encyclopedia of genes and genomes database; KOG: Eukaryotic clusters of orthologous groups; LSUrDNA: Large-subunit ribosomal DNA; NCBI: National center for biotechnology information; MAT: Mating-type; MCL: Markov cluster; MFS: Major facilitator superfamily; Myr: million years; NRPS: Non-ribosomal peptide synthetase; PCR: Polymerase chain reaction; PKS: Polyketide synthase; qRT-PCR: Quantitative real-time PCR; RIP: Repeat-induced point mutation; SEM: Scanning electron microscopy; SSUrDNA: Small-subunit ribosomal DNA; T6P: Trehalose-6-phosphate; TPS: Trehalose 6-phospate synthase; SSP: Small secret protein.

## Competing interests

There are no competing financial interests associated with this work.

## Authors’ contributions

YYW performed the biological assays, analyzed the genomic and qRT-PCR data and wrote the draft manuscript. BL was in charge of the genome sequencing and gene annotation. XYZ provided the analysis of genome data. QMZ took part in writing the draft manuscript, designed the biological experiments and analyzed the data. TZ isolated and determined the strain of lichen-forming fungus *E. pusillum* and its phycobiont *D. chodatii*, and optimized their culture for the study. HL provided the genes concerning sexual cycle of fungi and constructed two color figures (Figures 1 and 10). YFY and XLZ participated in the analysis of genome data. XYH and MW assisted with the genome sequencing. LW initiated the study and finalized the manuscript. JCW initiated and designed the study, wrote parts of the manuscript, and revised and finalized the manuscript. All authors read and approved the final version of the manuscript.

## Supplementary Material

Additional file 1 **Genomic analysis of *Endocarpon pusillum*.** The file contains additional information on genomic properties and qRT-PCR assays, comprising 10 tables provided in separate excel sheets. **Table S1** summarizes the main features of the primary sequence data. **Table S2** provides information on genes encoding proteins involved in sexual and asexual reproduction in *E. pusillum*. **Table S3** is a comparison of the number of secreted proteins between *E. pusillum* and other phytopathogenic fungi. **Table S4** lists the domain structures of predicted *E. pusillum* PKS and NRPS genes. **Table S5** lists the genes involving in drought resistant mechanisms in *E. pusillum*. **Table S6** provides information on genes involved in heterokaryon incompatibility in *E. pusillum*. **Table S7** lists the lectins measured by qRT-PCR analysis. **Table S8** lists genes that encode nitrogen transporters or proteins involved in nitrogen metabolism in *E. pusillum*, whose transcriptions were determined by qRT-PCR analysis. **Table S9** lists the homologous genes involved in symbiotic fungal sucrose and monosaccharide transporters in *E. pusillum*. **Table S10** lists genes that encode sugar transporters or proteins involved in sugar metabolism in *E. pusillum*, whose transcriptions were determined by qRT-PCR analysis. **Table S11** lists the candidate reference genes for qRT-PCR analysis in *E. pusillum*. Click here for file

## Accession number

The whole project has been deposited at DDBJ/EMBL/GenBank under accession number APWS00000000.
